# Aortic root rotation: morphological analysis of the aortic root with three-dimensional computed tomography

**DOI:** 10.1093/ejcts/ezae040

**Published:** 2024-02-03

**Authors:** Jules Miazza, David Winkel, Florian Thieringer, Oliver Reuthebuch, Friedrich Eckstein, Brigitta Gahl, Denis Berdajs

**Affiliations:** Department of Cardiac Surgery, University Hospital Basel, Basel, Switzerland; Department of Radiology, University Hospital Basel, University of Basel, Basel, Switzerland; Medical Additive Manufacturing Research Group (Swiss MAM), Department of Biomedical Engineering, University of Basel, Allschwil, Switzerland; Department of Oral and Cranio-Maxillofacial Surgery, University Hospital Basel, Basel, Switzerland; Department of Cardiac Surgery, University Hospital Basel, Basel, Switzerland; Department of Cardiac Surgery, University Hospital Basel, Basel, Switzerland; Department of Cardiac Surgery, University Hospital Basel, Basel, Switzerland; Department of Cardiac Surgery, University Hospital Basel, Basel, Switzerland

**Keywords:** Aortic root, Geometry of the aortic root, Aortic valve reimplantation, Three-dimensional modelling, Virtual surgical planning

## Abstract

**OBJECTIVES:**

The aortic root (AoR) rotation and its spatial morphology at the base of the heart were postulated but not described in every detail. AoR rotation modalities may play an important role in decision-making during AoR surgery and its outcome. The aim was to provide a detailed spatial anatomy of the AoR rotation and its relation to the vital surrounding structure.

**METHODS:**

The AoR rotation and its relation to the surrounding structure were assessed in 104 patients with tricuspid aortic valve. The interatrial septum was chosen as a reference to describe AoR rotation that marked the midline of the heart base as a landmark for the AoR rotation direction. Intermediate, clockwise and counterclockwise AoR rotations were defined based on the mentioned reference structures.

**RESULTS:**

The AoR rotation was successfully assessed in 104 patients undergoing ascending aorta and or AoR intervention by multidetector row computed tomography. AoR was positioned normally in 53.8% of cases (*n* = 56) and rotated counterclockwise in 5.8% (*n* = 6) and clockwise in 40.4% (*n* = 42) of cases. In clockwise AoR rotation, the right coronary sinus was positioned in proximity to the right atrium and of the tricuspid valve, whereas in a counterclockwise rotation, the noncoronary sinus was placed over the tricuspid valve just over the membranous septum.

**CONCLUSIONS:**

The AoR’s rotation can be diagnosed using multidetector row computed tomography. Understanding the anatomy of the aortic valve related to rotational position helps guide surgical decision-making in performing AoR reconstruction.

**Figure:**
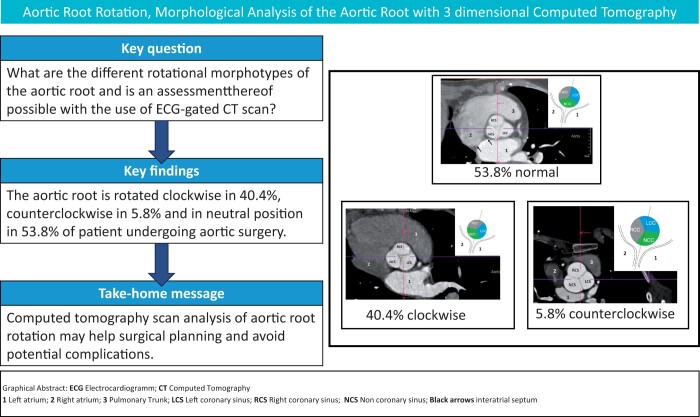

## INTRODUCTION

The aortic root (AoR) has a complex morphology, and its spatial architecture follows a well-defined pattern. The AoR is in close morphological relation to the surrounding structures, such as a mitral valve, tricuspid valve and heart conducting system. As such, it is a part of the complex anatomical relief at the base of the heart. Indeed, in past decades, AoR reparative procedures have evolved and are gaining increasing attention in the surgical community. Favourable mid- and long-term results [1–3] triggered interest in the procedure. Since the surgical approach is technically demanding, the focus has been on morphological characteristics of the AoR elements and their effects on surgical technique [4–6].

Although the AoR and its valvular apparatus create a complex architecture, its topography in relation to surrounding structures has not been discussed in detail. A previous study reported that right atrial perforation and tricuspid valve injury result from AoR proximity to the mentioned structures and troublesome dissection [7]. Furthermore, the manipulation of the AoR base was brought in direct causative relation to the postoperative conduction system damage [8]. Indeed, elements of the AoR base proved to have some diversity in relation to the right atrium, heart conducting system elements and the tricuspid valve [9]. In recent literature, a few reports focused on variations in the rotational position of the aortic valve. Different rotation variations may affect outcomes following transcatheter aortic valve implantation [10–14]. However, data analysis was conducted with a handful of data.

In this study, we conducted a retrospective analysis of all patients undergoing ascending aorta and AoR procedures and studied variations in the rotation of the aortic valve relative to the skeleton of the heart. The anatomical landmarks of the aortic valve and the heart skeleton based on electrocardiograph-gated multislice computed tomography (CT) scan images were analysed to evaluate the potential efficacy of preoperative diagnosis in assessing the risk of surgical complications.

## MATERIALS AND METHODS

### Ethics statement

The local ethical committee at the University of Basel, Basel, Switzerland (Ethikkomission Nordwest und Zentralschweiz, EKNZ 2021-02323) approved the protocol of this retrospective study. A written informed consent was waived due to the study’s retrospective nature.

### Study population

In the analysis, we included *n* = 104 consecutive adult patients with tricuspid aortic valves who underwent surgery for dilatative ascending aorta and/or AoR pathology between January 2015 and December 2020. Baseline patient characteristics and echocardiographic data were obtained from a review of medical records.

Patient records were selected and extracted from our institutional database (Intellect 1.7, Dendrite Clinical Systems, Henley-on-Thames, UK), which is checked for completeness and consistency monthly. AoR characteristics and aortic dimension size AoR base, sinus Valsalva, sinotubular junction and ascending aorta diameters were obtained from the electrocardiograph-gated multislice CT scan.

### Aortic root rotation assessment

**Figure 1:**
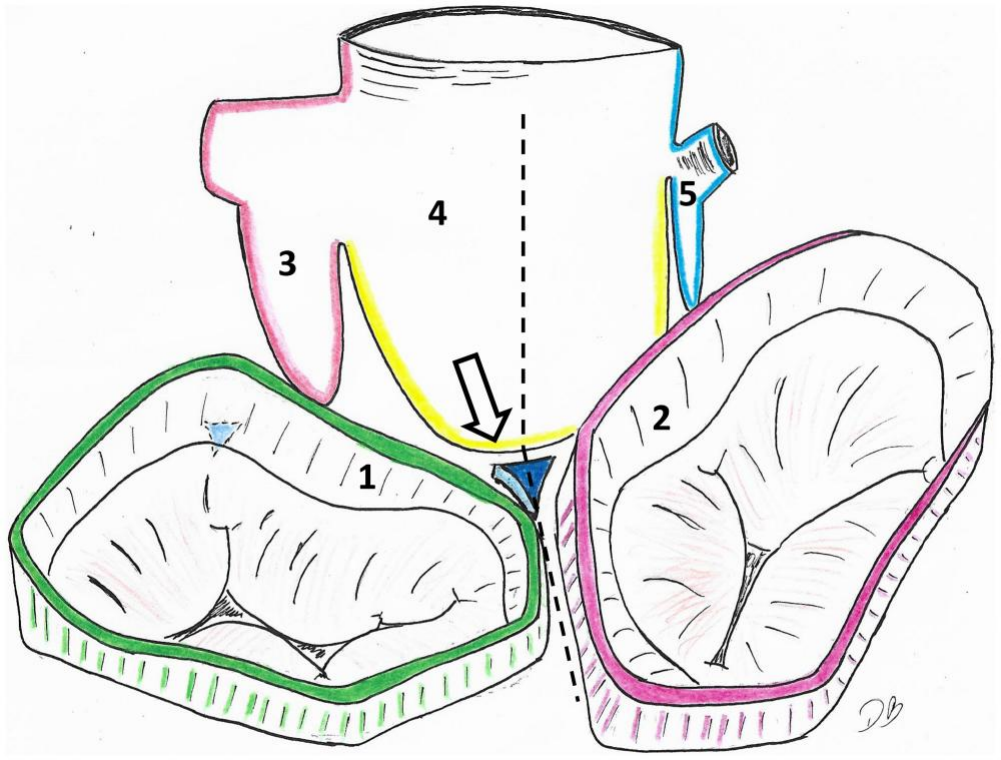
Schematic drawing of the heart base. The aortic root is seen from the posterior superior view. The left and right atrium were removed. The right fibrous trigonum is positioned just at the nadir of the noncoronary sinus. The dashed line marks the middle of noncoronary sinus, and the position of the interatrial septum. The arrow marks the position of the right intervalvular triangle. 1: left atrium; 2: right atrium; 3: left coronary sinus; 4: noncoronary sinus; 5: right coronary sinus.

**Figure 2:**
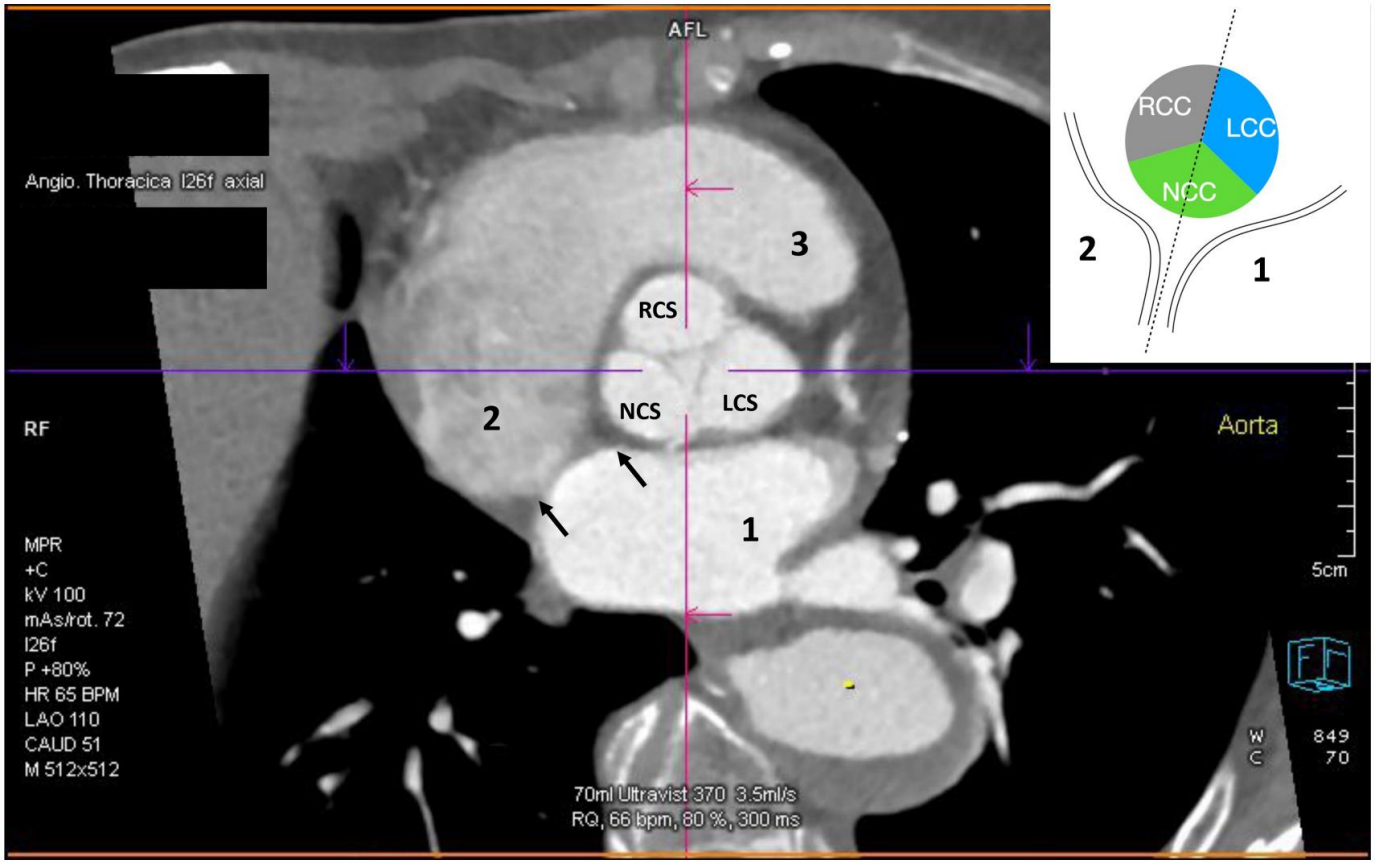
Electrocardiogram-gated multislice computed tomography scan of normal rotated aortic root. The arrows mark the position of the interatrial septum, which indicates the middle of the noncoronary sinus. The vignette schematic drawing presents aortic root relation to the interatrial septum. LCC: left coronary cusp; NCC: noncoronary cusp; RCC: right coronary cusp; 1: left atrium; 2: right atrium; 3: right ventricular outflow tract,

The heart skeleton’s elements were chosen as reference landmarks to assess the AoR rotation. The heart skeleton comprises several elements such as the left and right fibrous trigonum and the annulus of the anterior mitral valve leaflet. The right fibrous trigonum is in the middle of the noncoronary sinus nadir, indicating the middle of the noncoronary sinus. The right fibrous trigonum is near the membranous septum and the atrioventricular (AV) conducting system. The rim of the interatrial septum is, at its inferior part, attached to the right fibrous trigonum, and indicates the middle of the noncoronary sinus (Fig. 1). Consequently, the interventricular septum was chosen as a landmark for AoR rotation on the CT scan (Fig. 2).

### Computed tomography examination of the aortic root rotation

The AoR rotation was examined using cross-sectional images of preoperative CT scans viewed from the apex. However, when performing aortic valve surgery, the aortic valve is viewed from the aortic side, the opposite side of the CT finding.

The counterclockwise rotation appears to rotate clockwise and vice versa, which might be confusing. The clockwise rotation was defined as the rightward deviation of the aortic valve and the counterclockwise rotation as the leftward deviation*.* The rules in Euclidian two-dimensional space were followed. The rotation axis was in the middle of the AoR. As a reference, the anterior rim of the interatrial septum was defined. The inferior part of the interatrial septum is attached to the right fibrous trigonum, which is a part of the aortic skeleton. Consequently, the interatrial septum’s position may be considered a fix landmark. In the neutral position of the AoR, one-half of the noncoronary sinus is adjacent to the right atrium and one-half to the left atrium. The anterior commissure is positioned just behind the pulmonary trunk vis-à-vis the interatrial septum. The left commissure is in front of the left, and the right is in front of the right atrium.

In the counterclockwise rotation, defined as a leftward rotation of the AoR, the left commissure is rotated from the left anterior wall towards the left coronary fossa. The right commissure is rotated towards the interatrial septum.

The clockwise rotation was determined as the rightward rotation. In this case, the left commissure is rotated towards interatrial septum, and the right commissure towards the pulmonary trunk. In addition, the anatomical variabilities of the AoR and the spatial relation of the coronary vessels to the AoR were assessed.

### Statistical analysis

Categorical variables were summarized as counts and percentages, and continuous variables were summarized as median and interquartile range (IQR). All analyses were conducted using Stata 16.0 (Stata Corp LLC, Lakeway Drive, College Station, TX).

## RESULTS

### Baseline characteristics

Preoperative patients’ characteristics are presented in Table 1. The median age of patients was 69 (IQR 61–75) years, and 62% (*n* = 64) were females. Eight patients had diabetes (7.7%), and 7 (6.7%) had coronary three-vessel disease. Renal impairment was registered in 3 cases (2.9%) and a history of myocardial infarction in 6 (5.8%) patients. The incidence of New York Heart Association ≥III was observed in 12 (11.5%) and atrial fibrillation in 8 (7.7%) cases. The left and the right ventricular functions were preserved in all patients.

**Table 1:** Preoperative patients’ characteristics

*n* = 104	Descriptives
Age (years)	69 (61–75)
Female, *n* (%)	64 (62)
Diabetes, *n* (%)	8 (7.7)
Coronary vessel disease, *n* (%)	7 (6.7)
Peripheral artery disease, *n* (%)	5 (4.8)
History of stroke, *n* (%)	11 (11)
Renal impairment, *n* (%)	3 (2.9)
COPD, *n* (%)	10 (10)
History of MI, *n* (%)	6 (5.8)
Hypertension, *n* (%)	65 (63)
Hypercholesterolemia, *n* (%)	28 (27)
NYHA, *n* (%)	
II	29 (28)
III	9 (8.7)
IV	3 (2.9)
NYHA III or IV, *n* (%)	12 (12)
AF preop, *n* (%)	8 (7.7)
Current smoker, *n* (%)	27 (26)
Ejection fraction (median IQR)	60 (55–65)

Continuous variables are presented as median and interquartile range.

COPD: chronic obstructive pulmonary disease; IQR: Interquartile Range; MI: myocardial infarction; NYHA: New York Heart Association; SD: standard deviation.

The peri-operative data are presented in Table 2. In all patients, the ascending aorta was replaced. Concomitant procedures were performed in 30% (*n* = 32) of cases. A coronary bypass was performed in 13% (*n* = 13) of cases and aortic valve replacement in 43% (*n* = 35). In 32%, a biological prosthesis was used; in 1.92% (*n* = 2), a mechanical prosthesis was implanted. Aortic valve-sparing procedure with reimplantation technique was performed in 24% (*n* = 25) of cases.

**Table 2:** Surgical details

*n* = 104	Descriptives
Emergency, *n* (%)	21 (20)
EuroScore II	6.1 (3.1–10)
Perfusion time (min)	142 (110–187)
Aortic cross-clamp time (min)	102 (75–132)
Concomitant procedures, *n* (%)	
CABG	13 (13)
Mitral valve repair	4 (3.84)
Mitral and tricuspid valve repair	1 (0.96)
Aortic valve procedures	60
Aortic valve replacement, *n* (%)	35 (34)
Biological prosthesis	33 (32)
Mechanical prosthesis	2 (1.92)
Aortic valve reimplantation, *n* (%)	25 (24)
Composite graft implantation, *n* (%)	16 (15.4)
Aortic arch interventions, *n* (%)	79 (76)
Elephant trunk, *n* (%)	11 (11)
Total arch replacement, *n* (%)	3 (2.9)

Continuous variables are presented as median and interquartile range.

CABG: Coronary Artery Bypass Grafting.

Emergency ascending aorta replacements were performed in 20% (*n* = 21) of cases. All cases suffered from type A aortic dissection. The median EuroScore II of the cohort was 6.1% (IQR 3.1–10). The postoperative data are presented in Table 3. The median hospital stay was 9 (IQR 7.0–14) days. The incidence of postoperative stroke was recorded in 8 (7.7%), and the incidence of renal failure was registered in 5.8 (*n* = 6). The 30 days mortality was 9.6% (*n* = 10), *n* = 3 patients died following aortic dissection intervention, *n* = 2 due to postoperative multiorgan failure, *n* = 2 due to the disabling stroke and *n* = 1 patient died due to the prolonged biventricular heart failure.

**Table 3:** Postoperative outcomes

*n* = 104	Descriptives
30-Day mortality, *n* (%)	10 (9.6)
ICU stay (days)	4.0 (1.0–6.5)
Reoperation for bleeding, *n* (%)	8 (7.7)
MI, *n* (%)	0 (0)
Stroke, *n* (%)	8 (7.7)
Atrial fibrillation at discharge, *n* (%)	36 (35)
Permanent pacemaker, *n* (%)	3 (2.9)
Sternal infection, *n* (%)	3 (2.9)
Postoperative renal failure, *n* (%)	6 (5.8)
Renal substitution therapy, *n* (%)	3 (2.9)
Pulmonary infection, *n* (%)	11 (11)
MACCE, *n* (%)	16 (15)
Sepsis, *n* (%)	2 (1.9)
Hospital stay (days)	9.0 (7.0–14)

Continuous variables are presented as median and interquartile range.

ICU: intensive care unit; MACCE: major adverse cardiovascular event; MI: myocardial infarction.

### Computed tomography examination of the aortic root rotation

The AoR was positioned normally in 53.8 (56%), rotated clockwise in 42 (40.4%) and rotated counterclockwise in 6 (5.8%) cases.

In a normally rotated AoR (*n* = 56), the left intervalvular triangle of the AoR (corresponding to the left commissure) was positioned over the middle of the anterior leaflet of the mitral valve. Its position corresponds to the A2 segment of the mitral valve. The anterior commissure is behind the right ventricular outflow tract (RVOT), just in front of the right fibrous trigonum and interatrial septum. In normal rotation, the middle of the noncoronary sinus was placed over the right fibrous trigonum just in front of the interatrial septum. The right half of the noncoronary sinus was placed over the membranous septum directly over the tricuspid valve's anterior commissure (Fig. 2).

**Figure 3:**
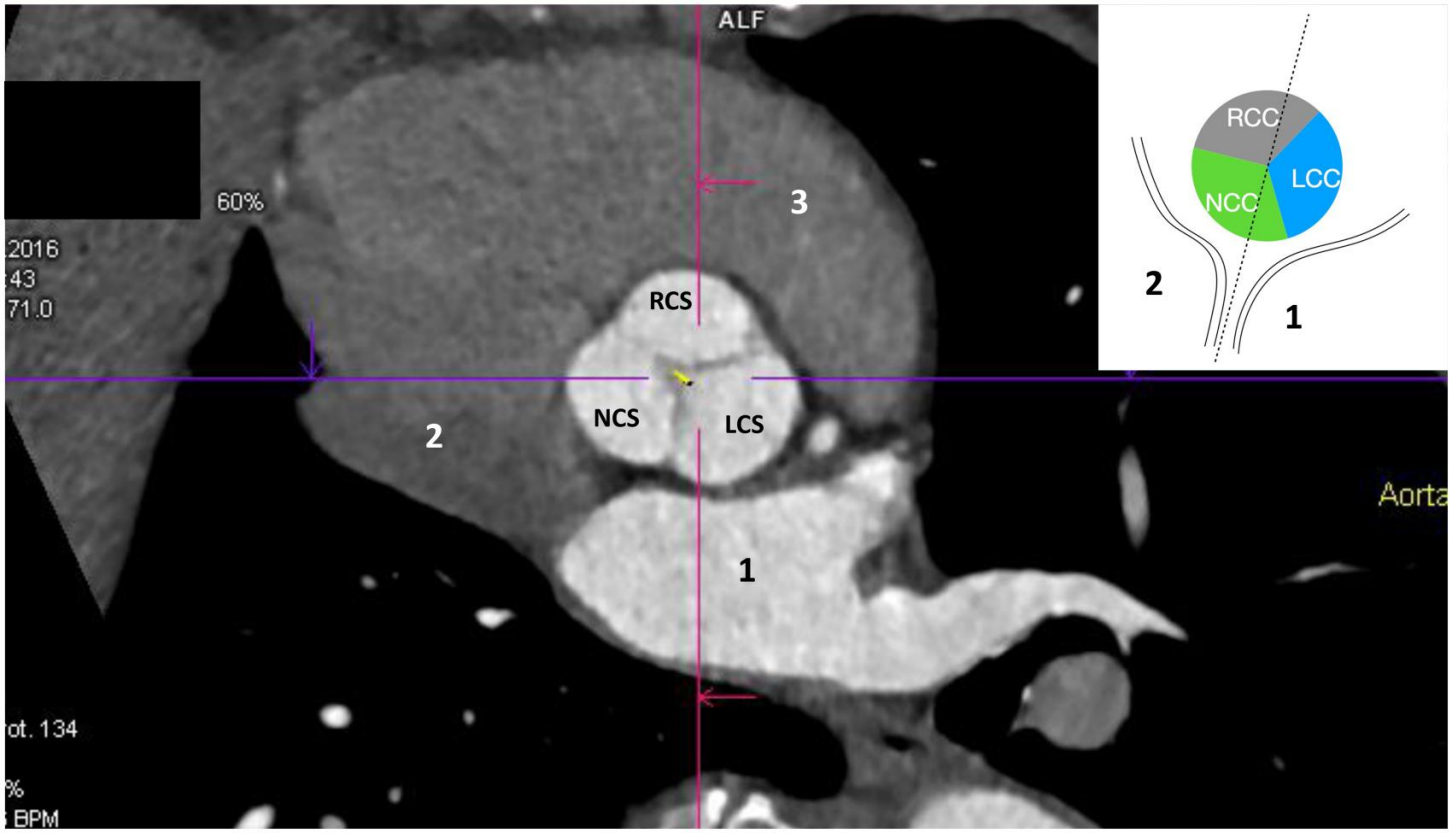
Electrocardiogram-gated multislice computed tomography scan of clockwise rotated aortic root. The vignette schematic drawing presents aortic root relation to the interatrial septum. LCS: left coronary sinus; NCS: noncoronary sinus: RCS: right coronary sinus; 1: left atrium; 2: right atrium; 3: right ventricular outflow tract.

In clockwise rotation (*n* = 42), the anterior commissure was rotated from the RVOT towards the left coronary fossa, a space between the posterior wall of the RVOT and the anterior wall of the left atrium. Consequently, the middle of the noncoronary sinus was rotated towards the anterior wall of the right atrium and tricuspid valve. This way, the left intervalvular triangle (corresponding to left commissure) and left half of the noncoronary sinus were positioned over the right fibrous trigonum in front of the interatrial septum (Fig. 3).

**Figure 4:**
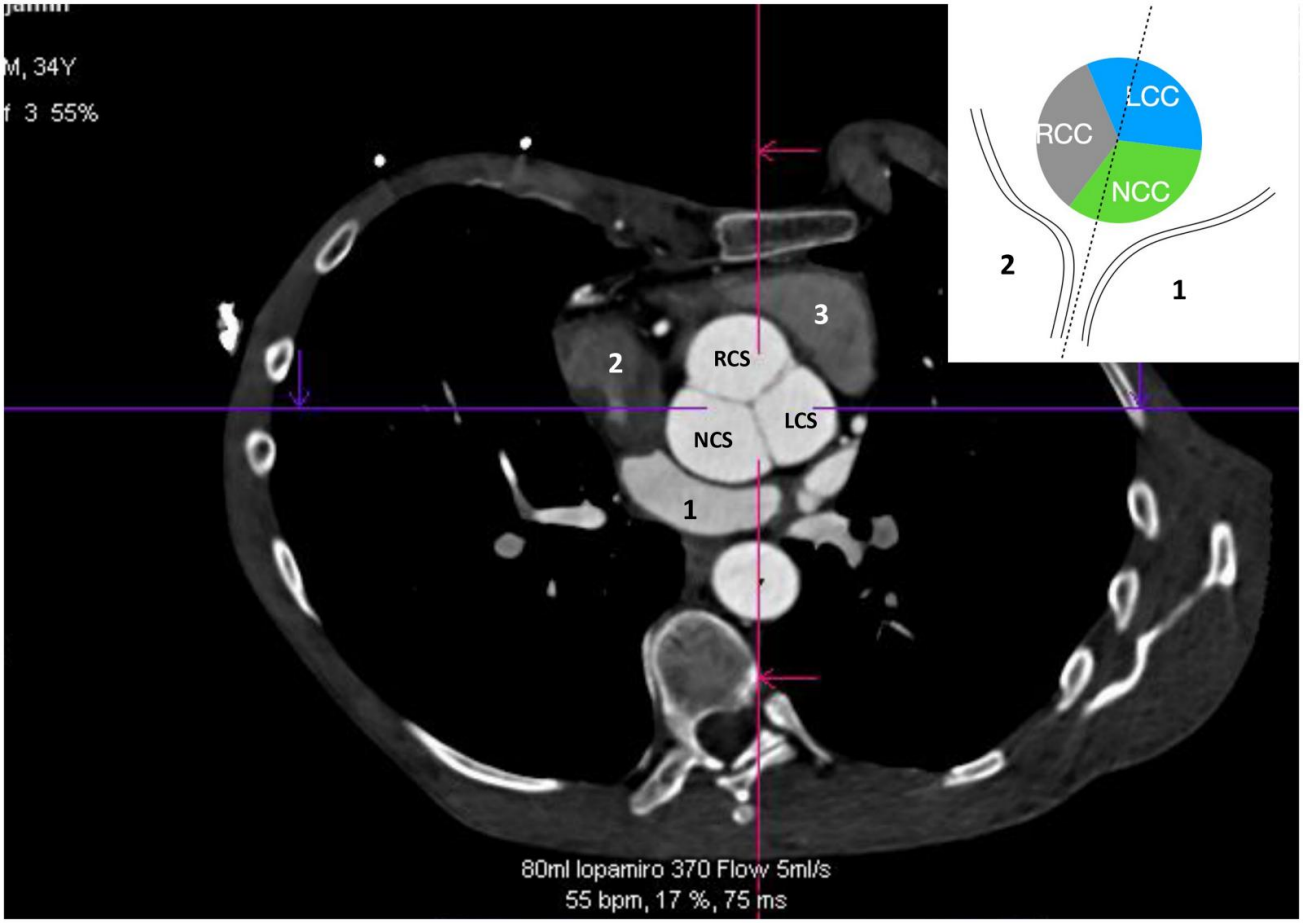
Electrocardiogram-gated multislice computed tomography scan of counterclockwise rotated aortic root. The vignette schematic drawing presents aortic root relation to the interatrial septum. LCS: left coronary sinus; NCS: noncoronary sinus; RCS: right coronary sinus; 1: left atrium; 2: right atrium; 3: right ventricular outflow tract.

In counterclockwise rotation, the left intervalvular triangle with corresponding commissure was rotated towards the coronary fossa and conjoined with the left fibrous trigonum. Consequently, the right half of the noncoronary sinus and the right commissure were in front of the interatrial septum. The middle of noncoronary sinus was due to the leftward rotation positioned near to the anterior leaflet of the mitral valve. The anterior intervalvular triangle was rotated towards RVOT, and the right coronary sinus was placed over the tricuspid valve in direct proximity to the heart conduction system (Fig. 4).

According to the AoR rotation, the coronary artery ostia were also displaced. In counterclockwise rotation, the ostium of the left coronary was displaced near the left intervalvular triangle and came in this variant next to the attachment of the aortic valve hinge area at the left commissure. The right coronary artery was displaced towards the anterior attachment of the right aortic valve leaflet.

In clockwise rotation, the left coronary ostium was near the anterior intervalvular triangle and anterior hinge of the left leaflet. In this case, the right coronary ostium was near the right intervalvular triangle.

## DISCUSSION

The rotation variants of the AoR were identified by analysing ECG-gated CT scans. Additionally, CT scan images identified structural elements at the base of the heart near the AoR that are important landmarks in determining the aortic root rotation. The interatrial septum, not an integral component of the AoR, is an important landmark for defining the AoR rotation. The interatrial septum is attached to the right fibrous trigonum in its inferior anterior part. The right fibrous with the left fibrous trigonum and the anterior annulus of the mitral valve form the heart’s skeleton, having a constant positioning in the heart structure. Unfortunately, to date, routinely applied image modalities do not allow visualization of the heart skeleton, so application of the fibrous trigonum as landmark in routine imaging is not possible. For this reason, alternative structural indicators were considered. The interatrial septum, which is directly linked to the right fibrous trigonum, was identified as a landmark in determining aortic root rotation.

In the neutral AoR position, the interatrial septum was in the middle of the noncoronary sinus. This variant was the most frequent, observed in almost more than half of the investigated cases. In the neutrally rotated AoR, the interatrial septum indicated the middle of the noncoronary sinus. Its right half was conjoined with the tricuspid valve’s membranous septum and anterior commissure. This corresponds to the anterior end of the Koch triangle, specifically the area of the AV node and its transition to the His bundle. In a neutral position, the left half of the noncoronary sinus was adjacent to the mitral valve and positioned over the A3 segment of the mitral valve. In the neutral AoR position, the triangle between the noncoronary sinus and the left coronary sinus was positioned over the middle of the anterior leaflet of the mitral valve, and the nadir of the left coronary sinus was attached to the left fibrous trigonum.

In counterclockwise rotation, the noncoronary sinus was rotated towards the tricuspid valve and RVOT. In this case, the nadir of the noncoronary sinus was placed over the septal leaflet of the tricuspid valve. The right commissure and intervalvular triangle were displaced anteriorly towards the RVOT and positioned superior to the anterior commissure of the tricuspid valve. The right intervalvular triangle is in direct continuation of the membranous septum, which is extended towards the anterior leaflet of the tricuspid valve. Consequently, the septal leaflet and the adjacent part of the anterior leaflet of the tricuspid valve were attached to the membranous septum.

On the other hand, the anterior displacement of the membranous septum may result in divergent topography of the AV conduction system. The His bundle is positioned over the muscular septum, running along the inferior edge of the membranous septum, where, in normal anatomical variants, it divides into the left and right branches at the level of the anterior commissure of the tricuspid valve. Extending the membranous septum towards the anterior leaflet of the tricuspid valve may also displace the His bundle and the conjoined conducting systems elements. Consequently, AoR interventions may expose the vulnerable AV node and His bundle elements. This is especially true when the AoR base is a subject of manipulation, such as in valve reimplantation or composite graft implantation. On the other hand, the intervention on the tricuspid valve could also be a risk factor for conduction system lesions in the left-sided AoR.

In recent studies, the CT scan has proven useful in diagnosing AoR rotation. In cross-sectional images, the rotation may be revealed by focusing on the position of the interatrial septum as one of the most important landmarks. Indeed, in daily practice, this finding may be overlooked without a detailed analysis of the AoR surrounding structures.

Besides the pure geometrical observation, additional morphological findings may be considered. For example, such as the width of muscular mass at the AoR base. This element was not quantified in recent analysis. However, qualitative image observation was consistent with our previous histological observations, for example at the right sinus of Valsalva and the left half of the left coronary sinus, the hinge area of the leaflet was embedded into the myocardial musculature. Especially in the right sinus of Valsalva, except for the segment superior to the membranous septum, the right musculature reached the inferior one-third of the sinus wall [15]. The myocardial musculature supported the whole right sinus in patients with right-sided rotation. This may be one of the unfavourable morphological elements when performing AoR reconstruction. This feature may be more pronounced in patients with a bicuspid aortic valve, where a large muscular segment is a part of the coronary sinus base called a sinking sinus [16]. In such cases, dissection of the AoR base for placement of the suture line at the AoR base may be accompanied by technical difficulties.

Another notable finding related to the AoR rotation is the displacement of the coronary arteries. The relationship between the leaflet attachment and the coronary artery ostium varies with AoR rotation modality. If the ostium is too close to the valve, it becomes difficult to detach the coronary artery and leave sufficient sinus wall tissue around its ostium to allow for reimplantation as a button. In those cases, various strategies may be employed, such as the VSRR procedure reported by Sheikh and David [17].

## CONCLUSION

This study characterized the anatomical variability of aortic valve rotations. Several pitfalls should be considered when performing AoR surgery, particularly in patients with a counterclockwise rotated aortic valve. First, the AV conduction system may have, depending on rotation variants, a different spatial relation to the established landmarks, such as in Koch triangle. That should be considered in reconstructive procedures involving manipulation of the AoR base. Thus, damage to the conduction system may be increased in counterclockwise AoR rotation, leading to the elevated incidence of AV conduction disorders. Furthermore, the area adjacent to the right ventricular membrane and of the tricuspid valve carries the risk of perforation as well of the right ventricle and tricuspid valve injury.

Furthermore, the origins of the coronary arteries may be displaced relative to the rotation of the aortic valve. To avoid intraoperative coronary injury, strategies for coronary reimplantation in root surgery need to be adjusted accordingly. We believe that detailed preoperative CT scan analysis may help predict possible complications and clarify the precautions to be taken during surgery.

## Data Availability

The data underlying this article will be shared on reasonable request to the corresponding author.

## ABBREVIATIONS

AoR: Aortic root
CT: Computed tomography
IQR: Interquartile range
RVOT: Right ventricular outflow tract
